# Impaired Inflammatory Response to LPS in Type 2 Diabetes Mellitus

**DOI:** 10.1155/2018/2157434

**Published:** 2018-01-14

**Authors:** Lusine Khondkaryan, Sona Margaryan, David Poghosyan, Gayane Manukyan

**Affiliations:** ^1^Group of Cell Technologies, Institute of Molecular Biology, National Academy of Sciences, Yerevan, Armenia; ^2^Laboratory of Molecular and Cellular Immunology, Institute of Molecular Biology, National Academy of Sciences, Yerevan, Armenia; ^3^Russian-Armenian (Slavonic) University, Yerevan, Armenia

## Abstract

Type 2 diabetes mellitus (T2DM) is a severe health problem worldwide, reaching epidemic levels. High susceptibility to infections of T2DM patients indicates dysregulated immune responses to pathogens. However, innate immune responses, including monocyte functions, in T2DM are poorly investigated. Therefore, in this study we aimed to assess lipopolysaccharide- (LPS-) induced immune responses of circulating monocytes from T2DM patients. The results showed that monocytes from T2DM were hyporesponsive to LPS challenge as reflected by significantly suppressed secretion of TNF*α* (*p* < 0.01) and expression of CD11b (*p* < 0.001) and TLR4 (*p* < 0.001) compared to those in monocytes from healthy subjects. Furthermore, LPS-induced IL-10 levels were similar in diabetic and healthy supernatants, while expression levels of CD163 were found to be downregulated on monocytes from T2DM (*p* < 0.001) suggesting impaired ability of monocytes to switch their phenotype to anti-inflammatory. Taken together, our results suggest compromised function of monocytes in T2DM, which may explain, at least partly, high incidence of infection in these patients.

## 1. Introduction

Type 2 diabetes mellitus (T2DM) represents a global public health concern with rapidly increasing prevalence worldwide [1]. It is a chronic metabolic disease characterized by a loss of insulin sensitivity by many cell types and progressive pancreatic *β*-cell dysfunction as a consequence of a hyperglycemia [2]. It has long been appreciated that chronic activation of the innate immune system is associated with T2DM. Growing evidence supports the involvement of inflammatory processes with an abnormal production of cytokines and activation of inflammatory signaling pathways in the development of this metabolic disease [3, 4]. Despite underlying chronic inflammation, high incidence of community-acquired and nosocomial infectious diseases in T2DM [5, 6] may indicate dysfunctions in innate immune response in these patients. Indeed, studies in T2DM subjects have revealed impaired phagocytic, chemotactic activity of monocytes and neutrophils [7–11], decreased natural killer cells and dendritic cell functions [12, 13], and so on. The presence of microbiota is associated with increased exposure of the host to microbe-derived products such as lipopolysaccharide (LPS). It was shown that a shift in the complex microbial system resulted in higher LPS levels in serum [14] and directly associated with the degree of insulin resistance [15].

Monocytes are the main cells of innate immunity that play central role in the initiation and resolution of inflammatory response to pathogens [16]. Recognition of LPS by its receptor TLR4 results in production of a broad range of molecules, including proinflammatory cytokines, chemokines, reactive oxygen species, and upregulation of adhesion and costimulatory receptors [17]. At the same time upregulation of anti-inflammatory mediators ensures successful resolution of the inflammatory response. A delicate balance between pro- and anti-inflammatory responses is required to prevent the progression from nonresolving acute inflammation to persistent chronic inflammation. Although monocytes are the main cells producing proinflammatory cytokines in norm, controversial results have been obtained concerning their potential role in inflammatory processes during T2DM [18–21]. Given the fact that LPS may abnormally activate monocytes, we aimed to determine whether circulating monocytes from T2DM patients exhibit differential features of activation after LPS challenge. For this, we assessed expression of surface markers TLR4, CD11b, and CD163 on circulating monocytes as well as production of TNF*α* and IL-10 by whole blood cells in the presence and absence of LPS.

## 2. Materials and Methods

### 2.1. Subjects

Peripheral blood samples were obtained from 10 patients with T2DM (mean age 53.2) and 7 healthy volunteers (mean age 41.8). Mean diabetes duration is 5.2 years. Patients were diagnosed at the “Surb Astvatsamayr” Medical Center (Yerevan, Armenia) based on criteria established by an expert committee on the diagnosis and classification of diabetes mellitus (1): (i) random venous plasma glucose ≥ 11.1 mmol/L, (ii) fasting plasma glucose ≥ 7 mmol/L, (iii) laboratory HbA1c > 48 mmol/mol (6.5%). All the patients with T2DM were treated with diet therapy and oral hypoglycemic agents. No patients received insulin therapy. None of healthy volunteers reported presence of diabetes or a family history of this disease, obesity, or chronic diseases. Study participant characteristics are given in Table 1. All participants in this study had provided written informed consent. The study was approved by the Ethics Committee of the Institute of Molecular Biology of the NAS RA (IRB IORG0003427).

### 2.2. Blood Sampling and Ex Vivo Stimulation of Whole Blood

The strategy for the whole blood assay was chosen to preserve physiological environment and avoid possible artificial stimulation of monocytes arising from mononuclear cell separation. Blood samples from all study participants were taken upon 12 h fasting, between 9:00 and 10:00 am. Venous blood was collected by venipuncture into a sterile tubes containing EDTA (Vacuette, Greiner Bio-One) and proceed within two hours. Aliquots of whole blood were diluted 1 : 3 in RPMI 1640 supplemented with 10% FBS, L-glutamine (2 mmol/l), and penicillin (100 U/ml) and cultured in the presence or absence of 100 ng/ml LPS (*Escherichia coli* 026:B6; Sigma) at 37°C for 24 hours. Afterwards supernatants were collected and stored at −70°C until assayed, while cells were harvested for the surface marker expression analysis.

### 2.3. Flow Cytometry

After stimulation, cells were harvested and contaminating red blood cells removed with lysis buffer. Remaining cells were washed with phosphate buffered saline (PBS) and incubated with following fluorescent monoclonal antibodies: CD14 PE-Cy7, TLR4 FITC, CD163 PE, and CD11b (activated) PE (BioLegend, UK) for 30 minutes in dark at room temperature. Appropriate mouse FITC, PE, and PE-Cy7-conjugated IgG subclasses were used as negative controls. Labeled cells after further washing with PBS supplemented with 0.5% BSA were fixed by the addition of fixation buffer (BioLegend, UK) and kept at 4°C until acquisition within 24 hours.

Samples were measured on BD FACScan flow cytometer (Becton Dickinson, San Jose, CA, USA) and at least 10000 events were obtained. The data was analyzed using FlowJo version 10.1 (Ashland, OR, USA). The monocyte population was determined as CD14 positive cells and back gated on FSC/SSC dot plot. The median fluorescent intensity (MFI) of TLR4, CD11b, and CD163 positive cells within monocytes population was estimated.

### 2.4. ELISA

The concentrations of TNF*α* and IL-10 in culture supernatants were measured by ELISA kits (Biolegend, UK), according to manufacturer's instructions. The detection limits were 7.8 and 3.9 pg/ml for TNF*α* and IL-10, respectively.

### 2.5. Statistical Analysis

Data analysis was performed with GraphPad Prism 5.01 (GraphPad Software, USA). Statistical significance between treatments (unstimulated versus stimulated) was analyzed by Wilcoxon matched-pairs test. Between-group differences were evaluated by Mann–Whitney *U* test. Significance was defined at the level of *p* < 0.05.

## 3. Results

### 3.1. LPS-Induced Surface Expression of Monocytes Markers

**Figure 1:**
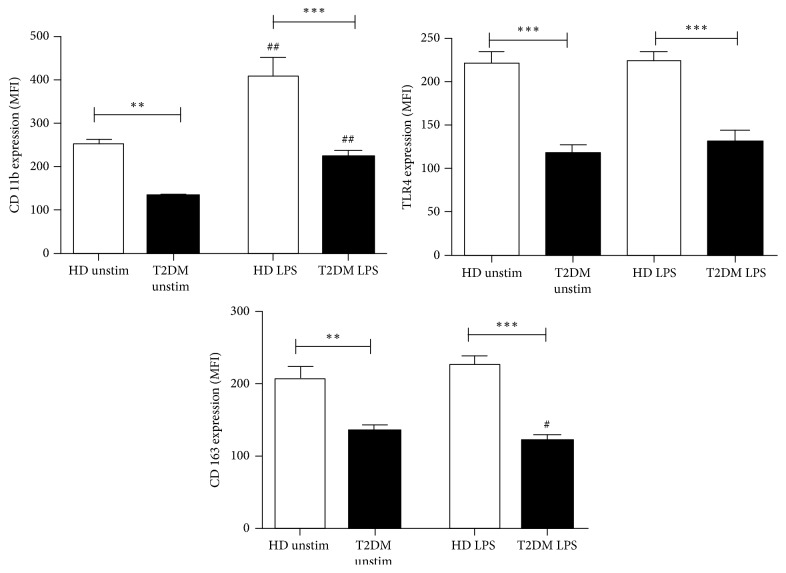
Surface expression of CD11b, CD163, and TLR4 on monocytes after cultivation of whole blood from healthy donors (HD, white bars) and type 2 diabetic (T2DM, black bars) patients with medium alone (unstimulated) or 100 ng/ml LPS for 24 hours. Data are given as median fluorescent intensity (MFI). Results are presented as mean ± SE. ^#^*p* < 0.05; ^##^*p* < 0.01 versus unstimulated cultures; ^*∗∗*^*p* < 0.01; ^*∗∗∗*^*p* < 0.001 versus healthy donors.

To evaluate the effect of LPS administration on expression of monocytes' surface receptors, whole blood was incubated with 100 ng/ml LPS for 24 hours and the median fluorescence intensity (MFI) of CD11b, TLR4, and CD163 were determined by flow cytometry. LPS treatment resulted in upregulation of CD11b expression on monocytes from both T2DM and healthy subjects (*p* < 0.01, Figure 1). However, the induction of CD11b was markedly decreased on diabetic cells, not reaching the expression levels on monocytes from healthy subjects (*p* < 0.001). Similarly, suppressed levels of CD11b were found on monocytes from T2DM patients cultured in medium alone (*p* < 0.01).

As shown in Figure 1, stimulation with LPS did not alter the expression levels of TLR4 in both studied groups. However, TLR4 expression was significantly reduced on both LPS-treated and unstimulated T2DM monocytes compared to respective cells from healthy donors (*p* < 0.001).

In order to evaluate the ability of diabetic monocytes to switch from proinflammatory towards anti-inflammatory phenotype, we measured the surface expression of CD163. LPS treatment resulted in decreased CD163 expression on monocytes from T2DM patients compared to unstimulated cells (*p* < 0.05, Figure 1). In contrast, monocytes from healthy donors slightly upregulated CD163 in response to LPS, though this increase did not reach statistical significance (*p* = 0.07). Respectively, the difference between LPS-treated CD163 was significantly lower in patients with T2DM as compared to healthy donors (*p* < 0.001).

### 3.2. LPS-Induced Cytokine Secretion

**Figure 2:**
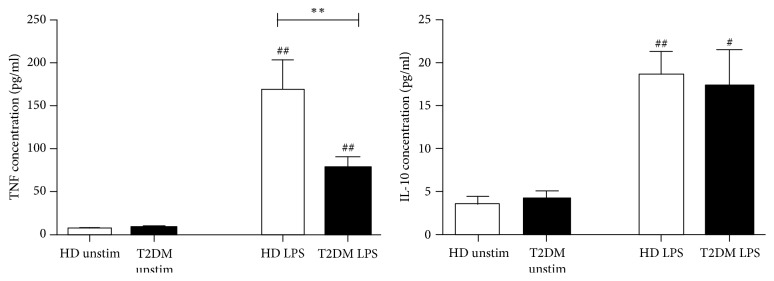
Concentrations of TNF*α* and IL-10 in supernatants after cultivation of whole blood from healthy donors (HD, white bars) and type 2 diabetic (T2DM, black bars) subjects with medium alone (unstimulated) or 100 ng/ml LPS for 24 hours. Results are presented as mean ± SE. ^#^*p* < 0.05; ^##^*p* < 0.01 versus unstimulated cultures; ^*∗∗*^*p* < 0.01 versus healthy donors.

Next, we studied TNF*α* and IL-10 production capacity by whole blood cultures of healthy and diseased subjects in response to LPS (Figure 2). LPS exposure resulted in significant increase in the secretion of TNF*α* and IL-10 in both groups as compared to unstimulated cultures (*p* < 0.01, Figure 2). Whereas concentrations of LPS-induced TNF*α* in T2DM were significantly lower than in healthy donors (*p* < 0.01), no differences in levels of IL-10 were found.

## 4. Discussion

Although chronic inflammation is one of the characteristics of T2DM, high incidence of infections in these patients may be partially attributed to disturbances in innate immune responses. In the present study, we clearly demonstrated dysregulation of peripheral blood monocytes in patients with T2DM. Monocytes from T2DM subjects exhibited initially impaired immune response as reflected by lower levels of phenotypic markers on the cells cultured in medium alone. Despite the fact that LPS exposure of monocytes from T2DM patients resulted in increased expression of CD11b and TNF*α* production, their levels were significantly lower than those in healthy controls. Observed “refractory state” of monocytes appeared to be a result of impaired LPS recognition, which is supported by the lower TLR4 expression on these cells. Failure of LPS to properly activate monocytes from T2DM patients in our study accounts for the endotoxin tolerance. This concept is further supported by the fact that low TNF*α* secretion was not counterbalanced by IL-10. It is likely that diabetic monocytes were primed by the low-dose* in vivo *LPS challenge, resulting in a reduced response of TNF*α* after* ex vivo* LPS stimulation. Indeed, recent evidences suggest that LPS play an important role in metabolic perturbations and might be a triggering factor for the onset of insulin resistance [15]. Low-grade increase in plasma LPS termed “metabolic endotoxemia” is a well-known feature of T2DM [22, 23]. On the other hand, T2DM is characterized as a state of hyperglycemia and hyperinsulinemia. Insulin has been shown to decrease monocyte TLR4 mRNA levels in T2DM [24], while high glucose levels have been reported to increase its membrane expression levels [25]. In parallel, it has been suggested that attenuated inflammatory response in T2DM is a direct consequence of hyperglycemia [26]. Taken together, hyperglycemia, hyperinsulinemia, and metabolic endotoxemia may affect monocytes functions in a complex manner. In contrast to our study, previous studies have demonstrated either increased or comparable levels of CD11b baseline expression in monocytes derived from T2DM patients [27–29]. Similarly, increased expression of TLR4 has been found in diabetic monocytes [25]. Intriguingly, Gupta et al. found suppressed TLR4 gene expression in diabetic patients with long disease duration and poor glycaemic control [30]. Methodological differences among studies that characterize the immune response may contribute to the discrepant findings.

Priming of innate immune cells by the endotoxin through TLR4 receptor enables the immune system to arise inflammatory response against potential pathogens. Fine-tuned regulation of excessive inflammation serves as an important mechanism for host protection against endotoxin shock [31, 32]. One of the classic examples of such protective mechanism is endotoxin tolerance [33]. However, when uncontrolled, this protective mechanism can become a serious clinical problem, increasing the risk for infection [34]. LPS-induced immune responses in T2DM have been a subject of considerable controversy. Attenuated, pronounced, or normal cytokine secretion in LPS-treated cells has been found in diabetic patients [18, 35–39].* Ex vivo* whole blood stimulation revealed conflicting results as well. No difference between diabetic and healthy subjects in the production of TNF*α* and IL-10 upon LPS treatment have been found [36, 40], while in study by Geerlings et al. [41] a tendency towards decreased secretion of TNF*α* has been reported. These discrepancies may be attributed to the differences in demographic and metabolic characteristics of studied participants. In our study lean healthy individuals were enrolled as a control group; however higher BMI in our patients could have diminished immune response [42].

In parallel, we analyzed the anti-inflammatory potential of diabetic monocytes by analyzing surface expression of CD163. Previously it has been shown that LPS treatment resulted in a rapid shedding of CD163 from monocyte surface (within 1 hour), followed by its upregulation (24 hour later) [43]. CD163 upregulation is a marker of switching from proinflammatory towards anti-inflammatory profile and IL-10 is one of the most potent stimulators of this process [43, 44]. Our results suggest that the ability of monocytes to change their phenotype towards anti-inflammatory is impaired in T2DM patients. Respectively, we observed decreased expression of CD163 on diabetic monocytes upon LPS exposure, while slight (albeit not significant) upregulation was observed on healthy cells. At the same time, IL-10 levels were comparable between groups, which suggest that monocytes from T2DM patients may be hyporesponsive to IL-10. Our data in an agreement with the study by Barry et al. [40] who showed that immunosuppressive effect of IL-10 is a result of impaired downstream IL10 signaling under hyperglycemic conditions. Contradictory results have been reported on the expression levels of CD163 on endotoxin-tolerant monocytes. While some studies suggested that upregulated CD163 on monocytes is a marker of endotoxin tolerance [45], the others found no association between expression levels and tolerant state [46]. Decreased expression of anti-inflammatory CD163 by monocytes in T2DM provides additional evidence that innate immune response in these patients is disturbed. Thus, impaired LPS-induced expression of the markers with apparent opposing functions could indicate exhausted phenotype of circulating monocytes as a result of their persistent activation.

The limitations of this study include small sample size and the fact that studied patients were treated with oral hypoglycemic agents which may interfere and affect results. Second, we showed only weakened response of monocytes to LPS in T2DM; however the direct association of this state with endotoxin dysfunction in an inflammatory environment has yet to be established. Our future research aims to address these limitations.

## 5. Conclusion

Our data suggest that monocyte function is impaired in T2DM patients, a finding which could alter inflammatory response during common infection. Failure to mount an adequate inflammatory response may represent a state of immunosuppression in these patients. Our results raise the question about the role of monocytes in pathogenesis and maintenance of low-grade inflammation in T2DM.

## Figures and Tables

**Table 1:** Participant characteristics.

	Healthy donors (*n* = 7)	Type 2 diabetes (*n* = 10)	*p* value
Gender (F/M)	4/3	8/2	
Age	41.8 ± 12	53.2 ± 7.8	NS
BMI (kg/m^2^)	20.9 ± 2.2	31.2 ± 4.7	0.001
Fasting blood glucose (mmol/L)	4.9 ± 0.77	8.3 ± 3.1	0.001

Data are means ± SD. *p* values correspond to the differences between healthy subjects and patients with T2DM.
